# Marginal Bone Level Measurements of Unsplinted Implants Used for Mandibular Overdentures: A Six-Month Randomized Prospective Clinical Study Comparing Early and Delayed Loading Protocols

**DOI:** 10.7759/cureus.35210

**Published:** 2023-02-20

**Authors:** Tarun Gaur, Roma Goswami, Apoorva Mowar, Deepika Sharma, Pushkar Gupta, Arpit Sharma, Neha Sharma, Anshul Saxena, Deepanshu Sharma

**Affiliations:** 1 Department of Prosthodontics and Crown & Bridge, Subharti Dental College, Meerut, IND; 2 Department of Oral and Maxillofacial Surgery, Subharti Dental College, Meerut, IND; 3 Department of Prosthodontics and Crown & Bridge, ITS (Institute of Technology & Science) Dental College, Muradnagar, IND; 4 Department of Prosthodontics and Crown & Bridge, Hitkarini Dental College & Hospital, Jabalpur, IND; 5 Department of Public Health Dentistry, Daswani Dental College & Research Center, Kota, IND; 6 Department of Conservative Dentistry & Endodontics, Modern Dental College & Research Centre, Jabalpur, IND; 7 Department of Prosthodontics and Crown & Bridge, Daswani Dental College & Research Center, Kota, IND

**Keywords:** marginal bone resorption, delayed loading, mandibular overdentures, early loading, implants

## Abstract

Background and purpose

Implant-supported mandibular overdentures are a good alternative for patients having poor retention of mandibular conventional dentures. The aim of this prospective study was to evaluate and compare the results between early loading and delayed loading of mandibular overdentures on two unsplinted implants.

Materials and methods

A total of 14 completely edentulous male patients in the age group of 50-60 years were selected for the study. Two 3.5×13 mm implants were placed in the mandibular interforaminal region. The patients were divided into two groups: (i) the test group in which the overdenture was connected after one week of surgery, and (ii) the control group, in which the overdenture was connected three months after surgery. Marginal bone levels were evaluated at baseline (during loading), three months, and six months post loading. Unpaired ‘t’ test was used for the comparison of intergroup measurements.

Results

No implants were lost. Marginal bone resorptions showed no statistically significant differences between the two groups over six months period after loading.

Conclusion

The results of this prospective clinical study suggested that there was no significant difference in the clinical and radiographic state of patients treated with implant-supported mandibular overdentures loaded either one week or three months after implant surgery.

## Introduction

For over a century, complete dentures have been the traditional standard of care for edentulous patients [1]. Although many edentulous patients are satisfied with their complete dentures, complaints occur in 10-30% of patients. They suffer from various problems, especially with the lower denture, such as insufficient stability, denture retention, and pain during biting [2]. In randomized and non-randomized clinical trials lasting six months to nine years, conventional dentures were shown to be inferior to two implant-retained mandibular overdentures. Patients find that their implant prostheses are significantly more stable and their ability to chew different foods is greatly facilitated [1].

Earlier, the recommended time between the placement of implants and their functional loading has been six to eight months in the maxilla and three to four months in the mandible [3,4]. However, the use of specially designed implants has reduced this time by creating greater bone-to-implant contact. According to the current literature, conventional loading is when the implants are allowed an osseointegration period of greater than two months before loading [5]. Early loading is when the implants are loaded between one week and two months [5]. Thus, the rationale for the study was to compare the outcome between early and delayed loaded implants so that the implants can be brought into function earlier than previously recommended. The aim of this study was to evaluate and compare the crestal bone loss between early and delayed loaded dental implants supporting mandibular overdentures in the interforaminal mandibular region.

## Materials and methods

The study was conducted in the Department of Prosthodontics, Subharti Dental College, Meerut, India, and was approved by the Institutional Ethical Committee of Subharti Dental College, Meerut, Uttar Pradesh, India (approval number: SDC/CER/2016). The study followed Equator guidelines and all the treatment was done according to the Helsinki declaration of 2013. Fourteen completely edentulous male patients in the age group of 50-60 years were selected for this study. The sample size was decided so that the trial can be completed within the limited time frame available for this academic study. Patients were informed regarding the procedure and written consent was obtained. Patients with health conditions such as bleeding disorders, uncontrolled diabetes, radiation therapy, cerebrovascular accident, myocardial infarction, valvular prosthesis surgery, pregnancy, immunosuppression, and ongoing treatment of malignancy were excluded from the study as they prohibit implant placement. Patients with pathological changes in the oral cavity, chronic smokers, neurological disorders, or any temporomandibular joint disorders were also excluded from the study.

Implant-supported mandibular overdentures on two unsplinted implants were constructed for all these patients. All the patients were first-time denture wearers. Tooth extractions were done between two months and one year before implant placement, having adequate inter arch space; the occlusal plane of the mandibular arch had approximately 10 mm space from the crest of the ridge to provide adequate space for the ball attachment. The patients were divided into two groups: Group 1 or the control group (delayed loading) and Group 2 or the test group (early loading) on the basis of primary stability obtained immediately after implant placement. Resonance frequency analysis (RFA) was done (Osstell®, Osstell AB, Göteborg, Sweden) to measure the primary stability obtained after implant placement. For safety reasons, patients with RFA values greater than 70 ISQ (implant stability quotient) were placed in the early loading group and patients with RFA values of 60-70 ISQ were placed in the delayed loading group. In Group 2 (early loading), the overdenture was loaded one week after surgery and in Group 1 (delayed loading), the overdenture was loaded three months after surgery. Balanced randomization of patients (7:7) was done in this parallel-group study to assess the equivalence of early loading with delayed loading.

Crestal bone loss was evaluated by intraoral periapical radiograph (IOPA), using a radiographic grid with the help of paralleling cone technique at baseline (during loading), three months, and six months post loading. 

Assessment of implant site

**Figure 1:**
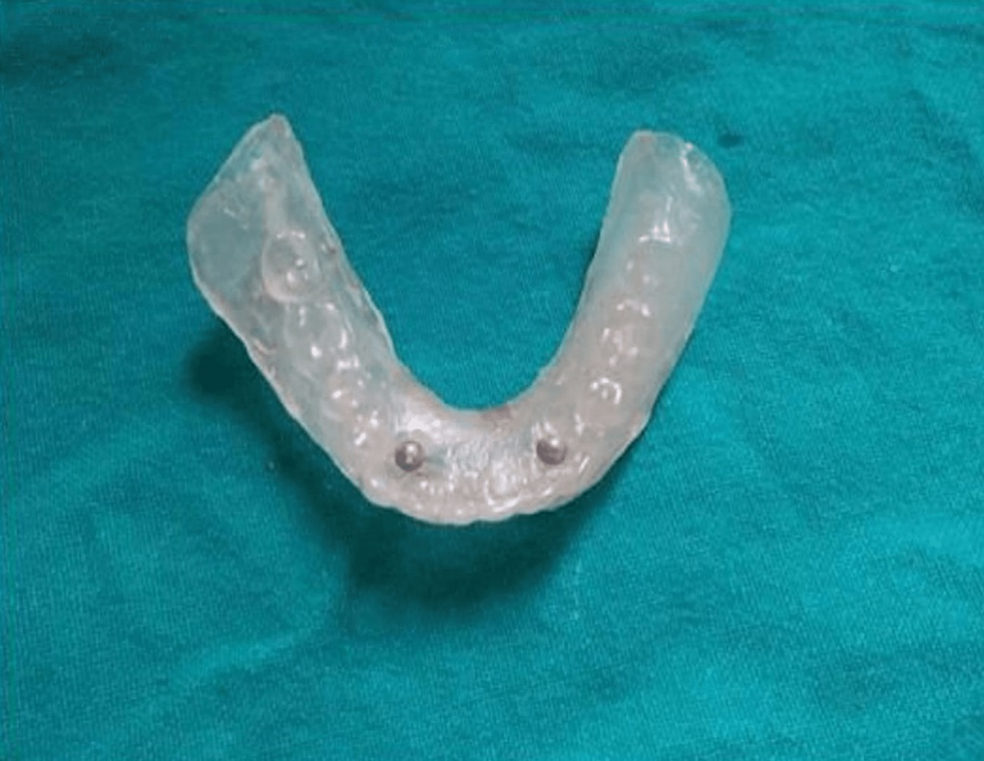
Radiographic template

The complete dentures were made according to a standardized protocol, which yielded optimal fitting and balanced occlusion [6]. The positions of the mandibular anterior teeth accorded with the neutral zone philosophy [7]. After the fabrication of conventional dentures, a radiographic template of clear autopolymerising resin was fabricated by duplicating the mandibular denture for the selected patients and two ball bearings were fixed in the mandibular canine region on both sides (sites B and D) (Figure 1). Once the template was in place, a cone beam computed tomography (CBCT) scan was performed and available bone width and height were measured using CBCT software.

Surgical procedure

**Figure 2:**
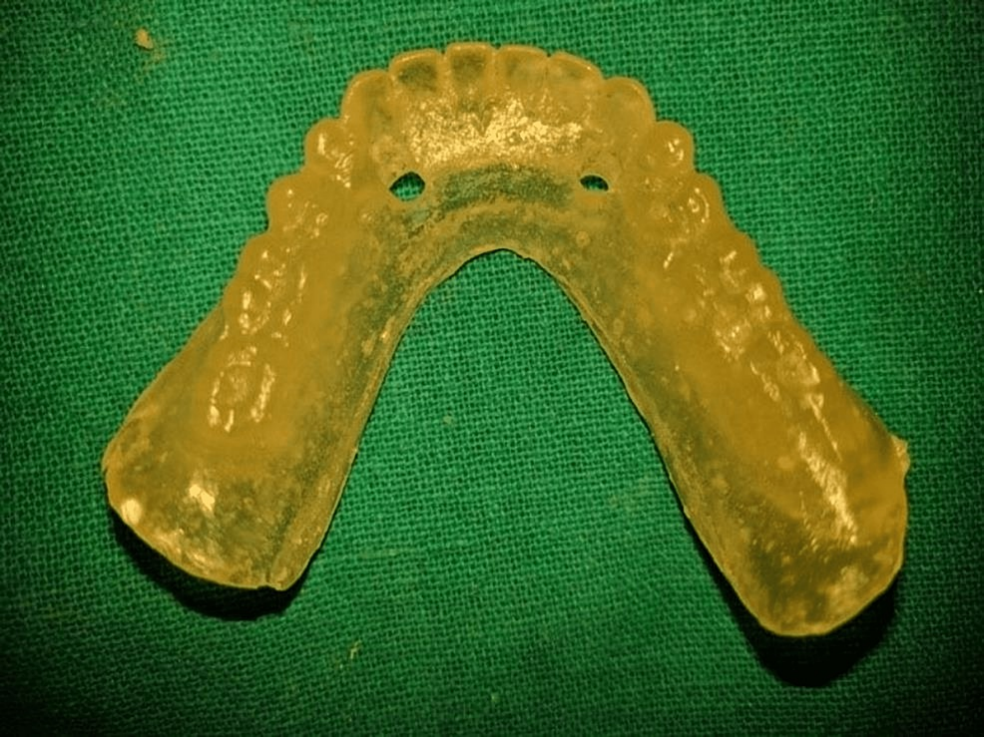
Surgical template

**Figure 3:**
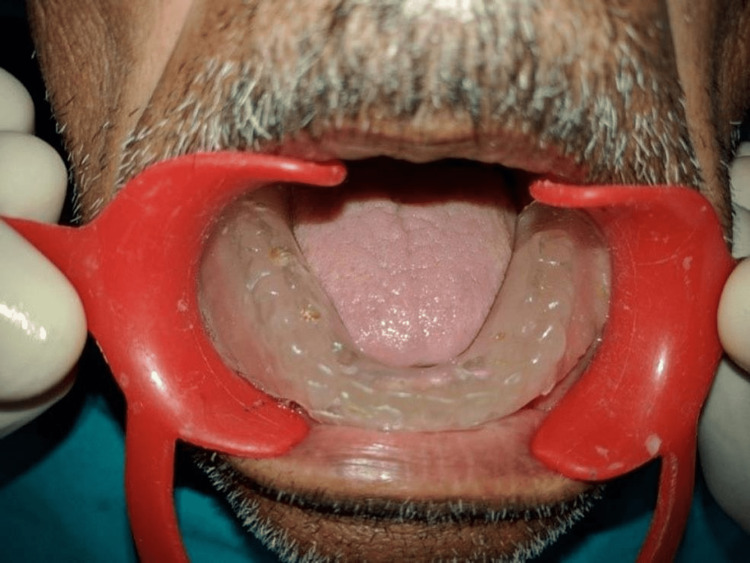
Surgical template in mouth

**Figure 4:**
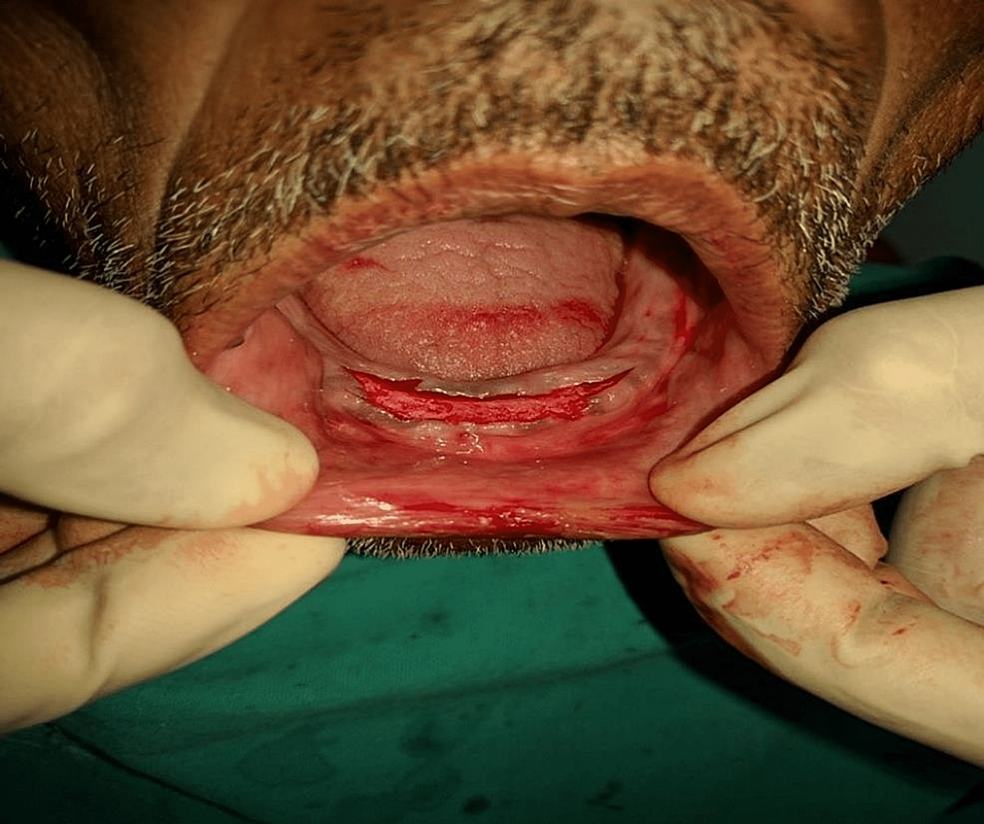
Crestal incision

**Figure 5:**
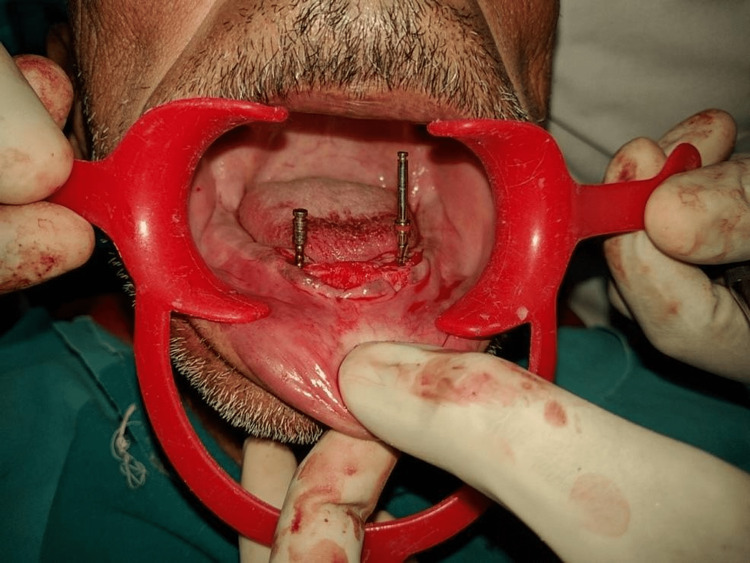
Paralleling pins placed

**Figure 6:**
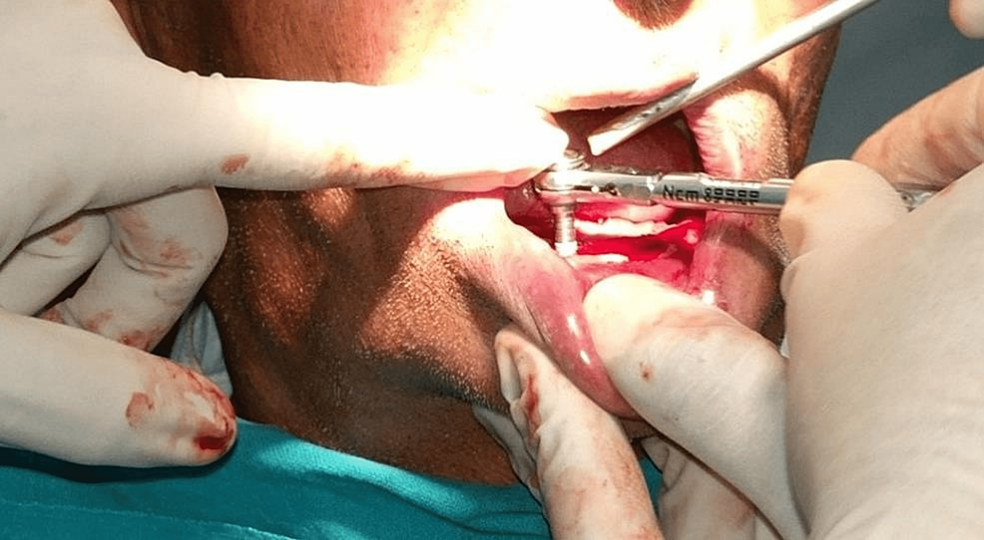
Implant placement using ratchet

**Figure 7:**
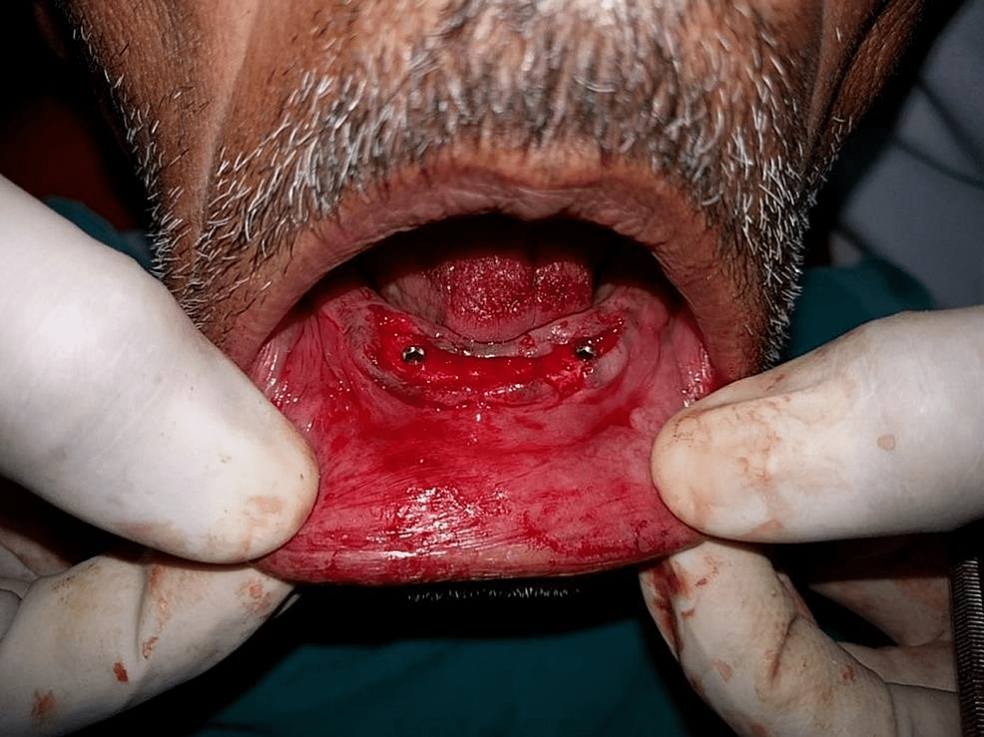
Implant placement done

**Figure 8:**
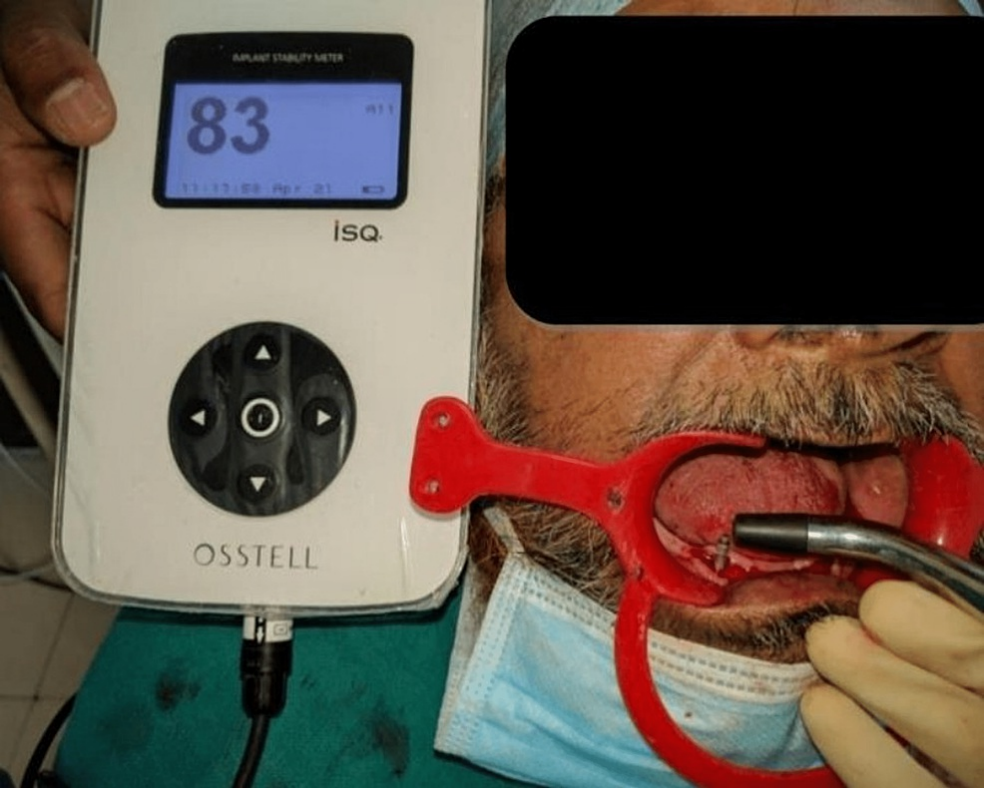
Resonance frequency analysis value taken after implant placement

All patients received oral antibiotics (Augmentin 625 mg three times a day) for five days, starting a day prior to the implant placement surgery. Two 3.5×13 mm implants (Touareg™-S, Adin Dental Implant Systems Ltd, Afula, Israel) were placed in each patient in the mandibular interforaminal region (B and D sites). The radiographic template was converted and utilized as a surgical template by drilling two 02 mm holes at the B and D sites (delineated by the point of attachment of the ball bearings) (Figures 2, 3). Implant placement surgery was carried out under local anesthesia using 2% lignocaine hydrochloride with epinephrine (1:20,000). Following the elevation of flap as in Figure 4, surgical stent was placed at the site of implant placement to guide the pilot drill to prepare an osteotomy site of appropriate length. After confirmation of depth and angulation as in Figure 5, the osteotomy site was prepared by a series of gradually wider drills (D2.8, D3.2) to the requisite width with a speed of 1200-1400 rpm at 1:20 reduction torque. Each implant was placed using a hand ratchet and ratchet adapter (Figure 6). Implants were placed 0.5-1 mm subcrestally (Figure 7). RFA value was taken after implant placement to evaluate the primary stability (Figure 8).

Prosthetic procedure

Group 1 (Conventional/Delayed Loading Group)

**Figure 9:**
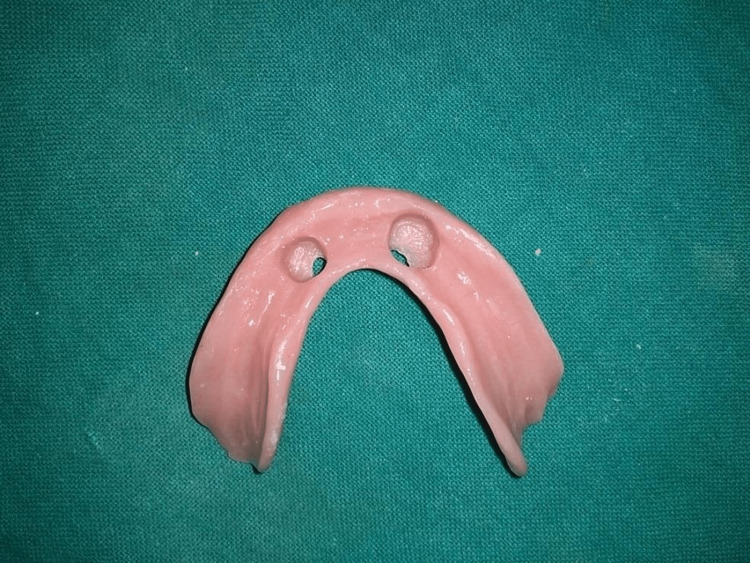
Hollowing out of the denture base and perforations made for the removal of excess autopolymerising resin

**Figure 10:**
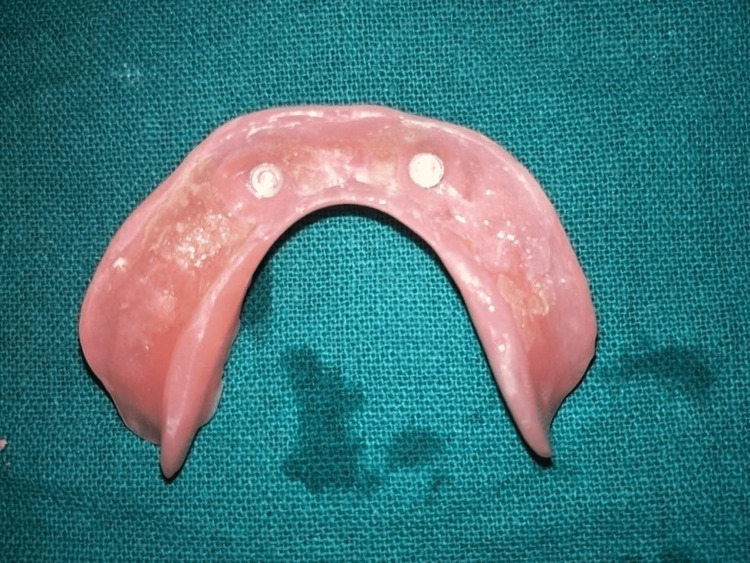
Metal housings and nylon caps after incorporation into the denture base

In patients belonging to this group, the implants were loaded three months postoperatively. Patients were recalled three months after implant placement and second-stage surgery was done to expose the implants, cover screws were removed, and ball abutments were placed. The metal housings and nylon caps were attached to the abutments. Articulating paper was used to transfer the position of metal housings to the denture base and the marked area was hollowed out (Figure 9). A pick-up procedure using autopolymerizing polymethyl methacrylate (PMMA) resin was used to attach the metal housings to the denture base (Figure 10).

Group 2 (Early Loading Group)

**Figure 11:**
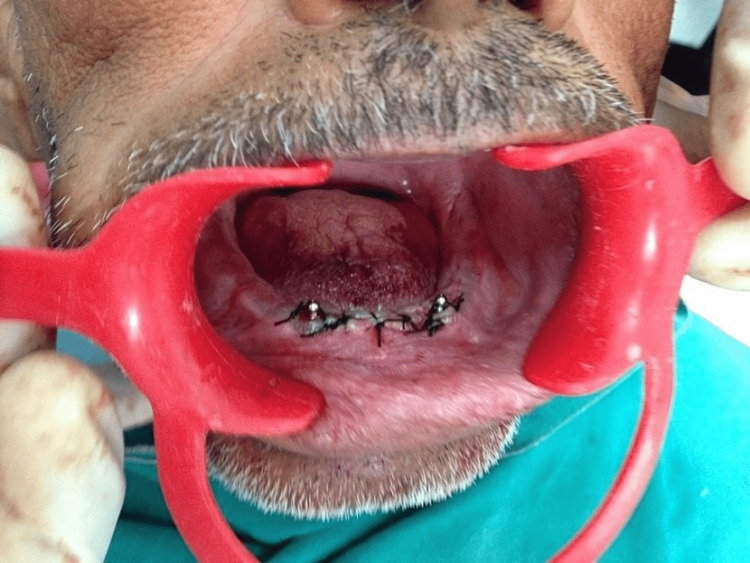
Ball abutments placed on the same day of implant placement and suturing done

**Figure 12:**
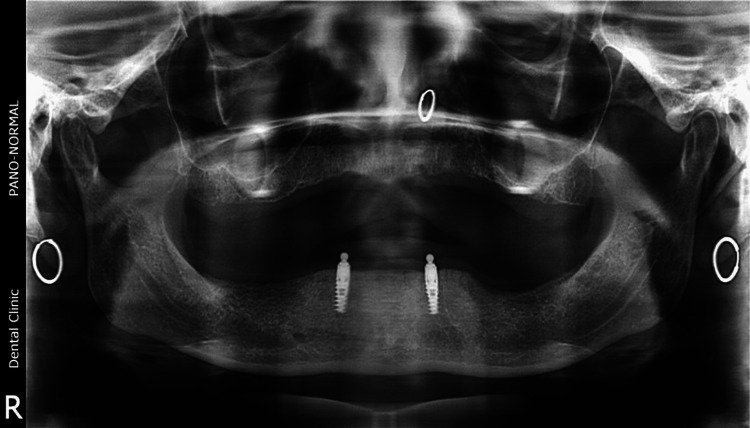
Orthopantomogram after implant placement

If high primary stability greater than 70 ISQ was obtained after implant placement, ball abutments were placed on the same day of implant placement as in Figure 11, sutures were placed, and a postoperative orthopantomogram (OPG) was taken (Figure 12). Patients were recalled on the seventh postoperative day. The metal housings and nylon caps were fitted to the denture similarly as in Group 1 and the implants were loaded on the seventh postoperative day.

Assessment of implant health

The following parameters were checked for all groups.

Implant Stability

The RFA device was used to assess the primary stability after implant placement. The RFA measures the resonance frequency with the help of a transductor attached to the implant body [8]. The measurement is denoted in terms of ISQ, which indicates the hardness of the connection between the implant and the bone [9]. ISQ values less than 45 indicate poor primary stability whereas ISQ values greater than 65 are considered most appropriate for implant stability [10]. The SmartPeg™ (Osstell AB, Göteborg, Sweden) was inserted into the implant, and reading was observed using an Osstell handpiece. The RFA handpiece was held perpendicular to the implant's long axis and the procedure was repeated three times for each assessment and averaged. According to the manufacturer, a value of 70 ISQ indicates that the implant is well osseointegrated and can be immediately loaded.

Crestal bone loss

A patient positioning device, a dental X-ray machine, and a 01 mm X-ray grid were used to assess proximal marginal bone loss. An electric potential of 65 kV and an exposure time of 0.1 seconds were maintained for each exposure. A radiographic grid was applied to the radiograph to facilitate the measurement of bone loss. All measurements were made on both the mesial and distal sides of the implant, from the junction of the implant and the abutment to the first bone contact with the implant. Based on these readings, mean bone loss was calculated for each patient. Measurements were made at the time of loading (baseline), three months, and six months post loading. A value of zero was noted if the bone level was found to be flush with the implant-abutment junction.

Statistical analysis

Statistical analysis was done from the data obtained. Mean and standard deviation (SD) were estimated for each patient and unpaired ‘t’ test was used for comparison of intergroup measurements.

## Results

None of the selected patients dropped out before the termination of the study and there were no postoperative complications in any patient.

Crestal bone loss

The crestal bone loss was measured on the mesial and distal aspect of each implant (B and D position) using a radiographic grid and mean values were calculated at baseline, three months, and six months post loading.

*Crestal Bone Loss at Site B* 

**Figure 13:**
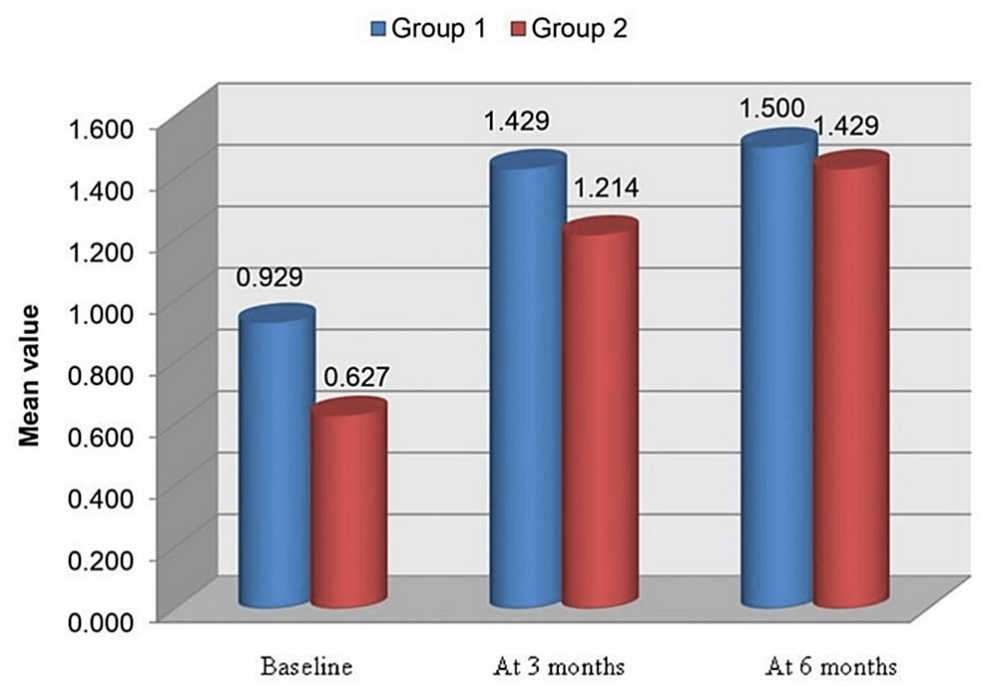
Crestal bone loss at site B (baseline, three months, and six months) for Group 1 and Group 2

In Group 1, the mean ± SD scores at baseline were 0.929±0.732, which increased after three months to 1.429±0.345. After six months, the mean values again increased to 1.500±0.408. There were no significant differences between the values at baseline, three months, and six months (Table 1, Figure 13). In Group 2, crestal bone loss scores at site B at baseline, three months, and six months were 0.627±0.237, 1.214±0.488, and 1.429±0.449, respectively. There were no significant differences between the values at baseline, three months, and six months (Table 1, Figure 13).

**Table 1:** Crestal bone loss at site B (baseline, three months, and six months) for Group 1 and Group 2 P<0.05: significant; P<0.001: highly significant

Time	Group 1 (n = 7)				Group 2 (n = 7)				Group 1 vs. 2
	Minimum	Maximum	Mean	SD	Minimum	Maximum	Mean	SD	P-value
Baseline	0.0	1.5	0.929	0.732	0.0	1.5	0.627	0.237	0.448
At 3 Months	1.0	2.0	1.429	0.345	0.5	2.0	1.214	0.488	0.361
At 6 Months	1.0	2.0	1.500	0.408	1.0	2.0	1.429	0.449	0.761

**Figure 14:**
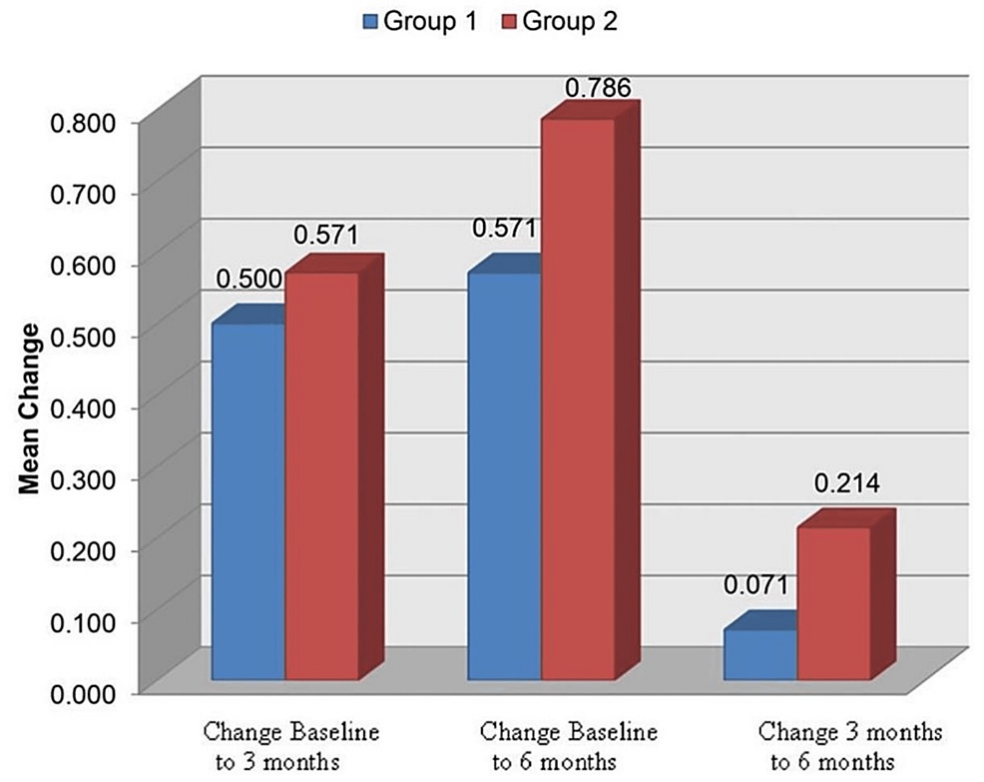
Comparison of change in crestal bone loss (site B) between groups (intergroup comparison)

On intergroup comparison of the change in the crestal bone loss at site B, using unpaired 't' test, it was found that there was no statistically significant difference between the two groups (Table 2, Figure 14).

**Table 2:** Comparison of change in crestal bone loss (site B) between groups (intergroup comparison using unpaired ‘t’ test) P<0.05: significant; P<0.001: highly significant

Time	Group 1 (n = 7)		Group 2 (n = 7)		Group 1 vs. 2
	Mean	SD	Mean	SD	P-value
Change Baseline to 3M	0.500	0.577	0.571	0.345	0.784
Change Baseline to 6M	0.571	0.534	0.786	0.488	0.449
Change 3M to 6M	0.071	0.189	0.214	0.393	0.403

Crestal Bone Loss at Site D

**Figure 15:**
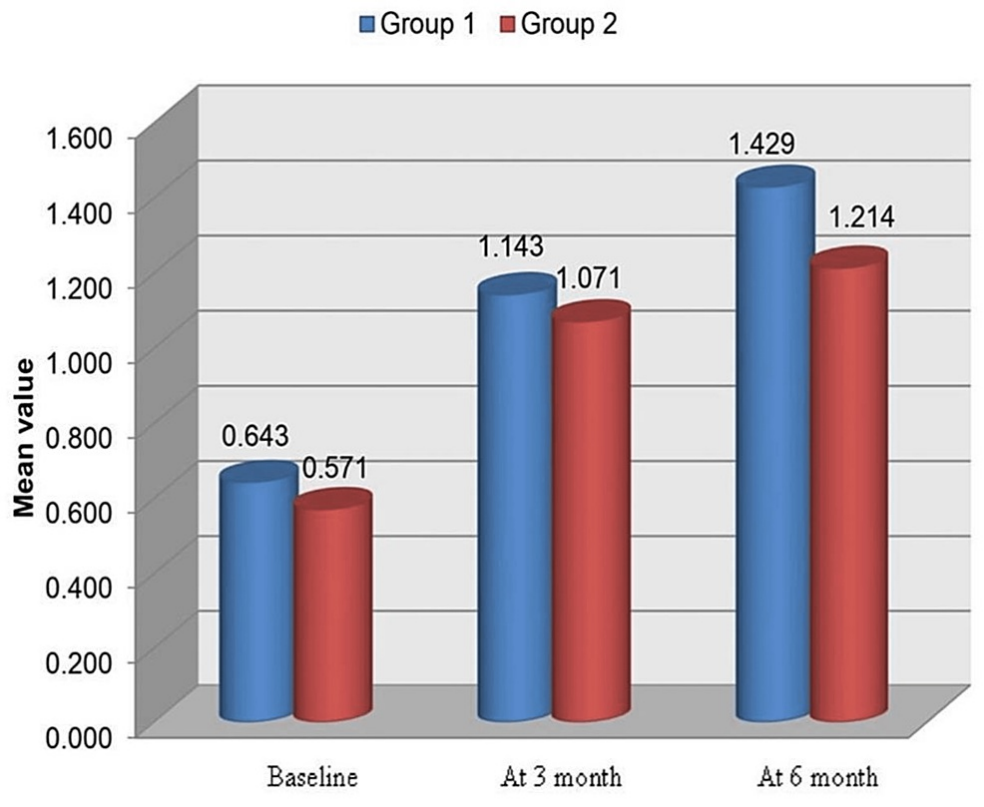
Crestal bone loss at site D (baseline, three months, and six months) for Group 1 and Group 2

In Group 1, the mean ± SD scores at baseline were 0.643±0.556, which increased after three months to 1.143±0.244. After six months, the mean values again increased to 1.429±0.345. There were no significant differences between the values at baseline, three months, and six months (Table 3, Figure 15). In Group 2, crestal bone loss scores at site D at baseline, three months, and six months were 0.571±0.345, 1.071±0.189, and 1.214±0.567, respectively. There were no significant differences between the values at baseline, three months, and six months (Table 3, Figure 15).

**Table 3:** Crestal bone loss at site D (baseline, three months, and six months) for Group 1 and Group 2 P<0.05: significant; P<0.001: highly significant

Time	Group 1 (n = 7)				Group 2 (n = 7)				Group 1 vs. 2
	Minimum	Maximum	Mean	SD	Minimum	Maximum	Mean	SD	P-value
Baseline	0.0	1.5	0.643	0.556	0.0	1.0	0.571	0.345	0.778
At 3 Months	1.0	1.5	1.143	0.244	1.0	1.5	1.071	0.189	0.552
At 6 Months	1.0	2.0	1.429	0.345	0.5	2.0	1.214	0.567	0.410

**Figure 16:**
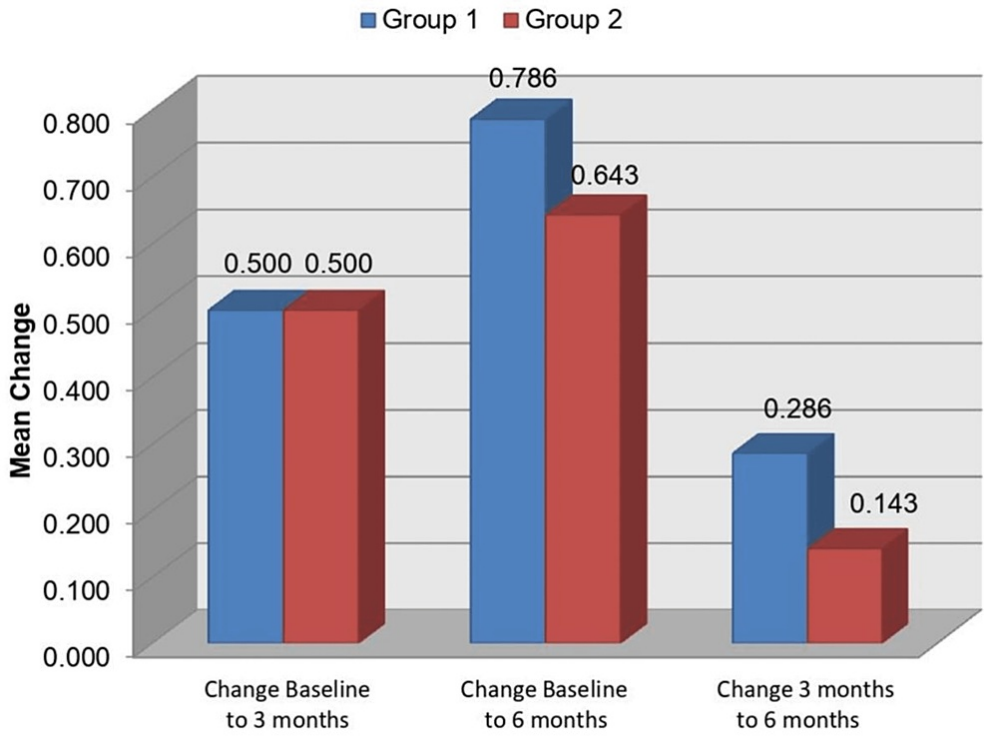
Comparison of change in crestal bone loss (site D) between groups (intergroup comparison)

On intergroup comparison of the change in crestal bone loss at site D, using unpaired 't' test, it was found that there was no statistically significant difference between the two groups (Table 4, Figure 16).

**Table 4:** Comparison of change in crestal bone loss (site D) between groups (intergroup comparison using unpaired ‘t’ test) P<0.05: significant; P<0.001: highly significant

Time	Group 1 (n = 7)		Group 2 (n = 7)		Group 1 vs. 2
	Mean	SD	Mean	SD	P-value
Change Baseline to 3 months	0.500	0.408	0.500	0.408	1.000
Change Baseline to 6 months	0.786	0.488	0.643	0.476	0.589
Change 3 months to 6 months	0.286	0.393	0.143	0.476	0.552

## Discussion

Crestal bone loss at site B

Group 2 presented with a mean crestal bone loss of 0.6 mm, 1.2 mm, and 1.4 mm at baseline, three months, and six months post loading, respectively. Group 1 presented with a mean bone loss of 0.92 mm, 1.42 mm, and 1.5 mm at baseline, three months, and six months post loading, respectively at site B. The values obtained from Group 1 (mean-1.28 mm) are slightly more than the values for Group 2 (mean-1.06 mm); however, no significant differences were observed between the two groups at site B. In our study, the maximum bone loss observed at site B in both groups at the end of six months was 1.5 mm, defined as the maximum amount allowed according to the success criteria given by Albrektsson et al. [11]. Turkyilmaz et al. observed an average bone loss of 1 mm after one year of loading [12]. There was no significant difference in the change in bone loss from baseline to three months, baseline to six months, and from three months to six months between the two groups at site B.

Crestal bone loss at site D

Group 2 presented with a mean crestal bone loss of 0.571 mm, 1.071 mm, and 1.214 mm at baseline, three months, and six months post loading, respectively. Group 1 presented with a mean bone loss of 0.643 mm, 1.143 mm, and 1.429 mm at baseline, three months, and six months post loading, respectively at site D. At site D also, the mean bone loss observed in Group 2 was less (0.952 mm) in comparison to Group 1 where the mean bone loss observed was 1.071 mm. There was no statistically significant difference between the two groups from baseline to three months, baseline to six months, and from three months to six months at site D.

Sekar et al. conducted a study in which they reported similar results, with more bone loss in the delayed loading group than the early loading group [13]. Higher bone loss could be attributed to inadequate oral hygiene maintenance by the patients.

Immediate/early loading of implants can be done if high primary stability is obtained. High primary stability can be obtained with the introduction of newer implants with improved surface characteristics; rough implant surfaces increase the bone-to-implant contact area. Implant diameter also influences implant stability [14]. With the help of improved surface characteristics, implants with diameters less than 3 mm could be placed with high primary stability in areas with minimal bone volume like in anterior ridges [15]. Almost all authors try to place implants of longer lengths wherever possible with a minimum 8 mm length, and the most frequently used were 13 and 15 mm [16]. 

Ball attachments were used in this study. According to an in vitro study conducted by Tokuhisa et al., the ball attachments were found to be more effective in minimizing denture movements and optimizing stress in mandibular implant-supported overdentures [17]. Sadowsky reported that ball attachments are less costly in comparison to other attachments and are less technique sensitive [18]. Locator attachments could also be used as an alternative to ball attachments as they provide good retention and higher patient satisfaction [19]. The minimum space required for locator attachments is also less, which is 8.5 mm, as compared to ball attachments, which require 10-12 mm of space [19]. However, no significant difference was observed between the attachment systems as reported in previous literature [19,20]. It appears that the success rate of implants does not get influenced by the attachment systems. Other factors like arch morphology, bone quality, and quantity seem to play far more important roles in the survival rates of implants [20,21]. However, further studies are still needed comparing different attachment systems used in implant overdentures.

Limitations

The small sample size of patients and the limited duration of the follow-up period, which was only six months after loading, were the primary limitations of this study. Though periapical radiography has been employed in numerous former studies, it cannot be considered unerring as it precludes facial and lingual bone level measurements, which might be considered inversely important in determining implant success.

## Conclusions

The results suggested that the conventional/delayed loading group showed a slightly higher marginal bone loss as compared to the early loading group. Poor oral hygiene must be associated with more bone loss. However, the difference between the two groups was not statistically significant.

Within the limitation of this study, the clinical outcome assessed was that the early loading of implant-supported mandibular overdentures could be considered a viable alternative treatment to the classic delayed protocols with faster patient rehabilitation in cases of high primary stability. More accurate long-term studies and large sample sizes are required to allow meaningful comparisons and conclusions.
